# Enhancing Primary Care Recognition of Type 1 Diabetes in Children: Diagnostic Challenges and Strategies to Prevent Diabetic Ketoacidosis

**DOI:** 10.3390/jcm15020533

**Published:** 2026-01-09

**Authors:** Yung-Yi Lan, Rujith Kovinthapillai, Andrzej Kędzia, Elżbieta Niechciał

**Affiliations:** 1 Center for Medical Education in English, Poznan University of Medical Sciences, 41 Jackowskiego St., 60-512 Poznań, Poland; catherine974u@gmail.com (Y.-Y.L.); rujithk22@icloud.com (R.K.); 2 Department of Pediatric Diabetes, Auxology and Obesity, Poznan University of Medical Sciences, ul. Szpitalna 27/33, 60-572 Poznań, Poland; akedzia@ump.edu.pl

**Keywords:** type 1 diabetes, diabetic ketoacidosis, pediatric diabetes, red flag symptoms, early diagnosis, practical guide, primary care clinicians, autoantibodies

## Abstract

Timely recognition of type 1 diabetes (T1D) in children and adolescents is crucial to prevent acute complications such as diabetic ketoacidosis (DKA). This narrative review examines the pathophysiology, clinical presentation, and diagnostic challenges of childhood T1D, including the young age of onset, clinician training gaps, and overlapping symptomatology between T1D and other common pediatric illnesses. Despite increased awareness, a significant proportion of children still present with DKA at diagnosis due to misinterpretation of symptoms, such as polydipsia, polyuria, and weight loss. This work emphasizes the importance of early recognition, timely intervention, and the use of structured management algorithms for primary care clinicians. Strategies to reduce DKA incidence, based on existing literature, successful real-world examples, and current guidelines, include enhanced screening for high-risk populations, educational initiatives, and improved diagnostic protocols. By implementing systematic approaches and public health campaigns, healthcare providers can improve early T1D detection and prevent severe DKA complications, ultimately enhancing patient outcomes and reducing long-term morbidity.

## 1. Introduction

Type 1 diabetes (T1D) is one of the most common endocrine disorders in children, characterized by hyperglycemia due to the autoimmune destruction of pancreatic islet β-cells [1,2,3]. Approximately 1.2 million children and adolescents under the age of 20 are affected globally, with the highest increases observed in the African, Middle East, and North African regions [4].

**Figure 1:**
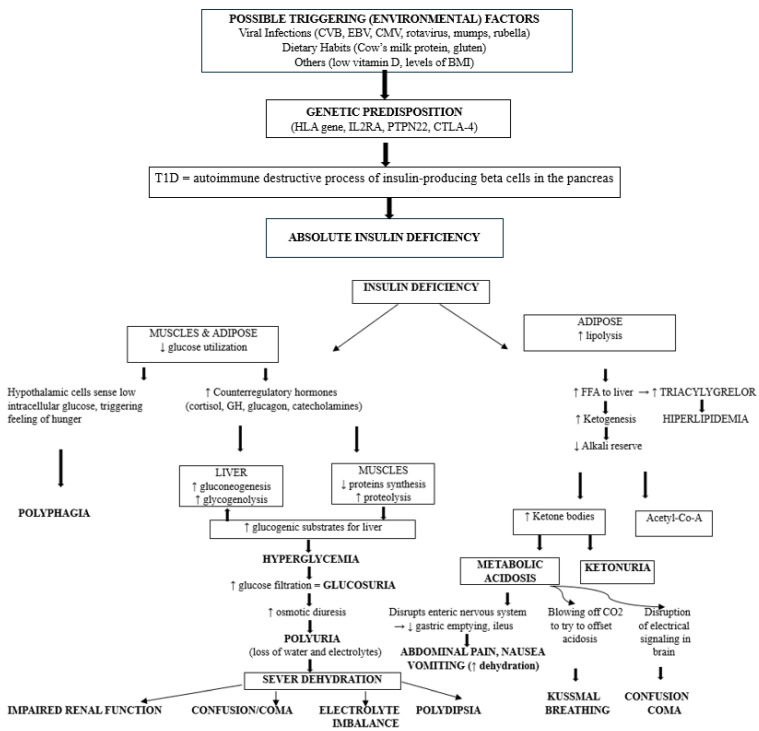
A flow diagram delineating the metabolic cascade triggered by an absolute insulin deficiency secondary to autoimmune pancreatic β-cell destruction in T1D, culminating in diabetic ketoacidosis and its characteristic clinical features [1,2,3,5,6,7,8]. CVB—Coxsackievirus B; EBV—Epstein-Barr Virus; CMV—Cytomegalovirus; BMI—Body Mass Index; *HLA*—Human Leukocyte Antigen; IL2RA—Interleukin-2 Receptor Alpha; PTPN22—Protein Tyrosine Phosphatase Non-Receptor Type 22; CTLA-4—Cytotoxic T-Lymphocyte Antigen 4; FFA—free fatty acid; Acetyl-CoA—acetyl coenzyme A; ↑—increase; ↓—decrease; →↓—no change or decrease.

Diabetic ketoacidosis (DKA), a potentially life-threatening complication, commonly presents at the childhood onset of T1D. DKA is marked by hyperglycemia [blood glucose ≥ 200 mg/dL (≥11.0 mmol/L), metabolic acidosis (pH < 7.3 or serum bicarbonate < 18 mmol/L), and elevated serum ketones (blood ß-hydroxybuyrate ≥ 3 mmol/L) or ketonuria (≥2+)] [5,6,7,8]. These biochemical derangements reflect absolute insulin deficiency; if unrecognized or untreated, they could trigger osmotic diuresis, severe dehydration, electrolyte disturbances, and cerebral metabolic stress, explaining why delayed diagnosis can rapidly progress to severe and life-threatening complications [9,10,11,12]. Figure 1 illustrates the downstream metabolic consequences of an absolute insulin deficiency resulting from the destruction of autoimmune pancreatic β-cells in T1D. The exact trigger is unknown, with strong hypotheses pointing to a combination of genetic susceptibility (*HLA* genes) and environmental factors, such as viral infections, dietary habits, among others, which may initiate autoimmunity in predisposed individuals. It shows how the cascade of insulin deficiency leads to hyperglycemia, ketogenesis, and progressive dehydration, the core biochemical features of DKA, and also provides a detailed explanation of the origin of its typical warning signs at the onset of T1D, such as polyuria, polydipsia, abdominal pain, and Kussmaul breathing [1,2,3,5,6,7,8].

Early identification of T1D is crucial for preventing DKA, which can develop rapidly after the onset of hyperglycemia. During T1D diagnosis, certain signs and symptoms can serve as red flags, indicating the need for prompt medical evaluation and intervention. Key red flags, including excessive thirst, frequent urination, and unexplained weight loss, however, are often overlooked, delaying diagnosis. Research indicates that a substantial proportion of children and adolescents present with DKA at the initial manifestation of the disease, underscoring the urgent need for increased awareness among healthcare providers and caregivers regarding the early signs of diabetes [13,14].

Globally, the prevalence of DKA at the onset of T1D varies substantially, with reported rates ranging from 14.7% to 79.8% [15]. Consistent with this global variability, up to three-quarters of children with new-onset T1D present with DKA at diagnosis, particularly among children at a younger age, specific ethnic backgrounds, and those experiencing diagnostic delays [16,17,18,19,20]. Beyond its high prevalence, DKA at T1D diagnosis places a substantial burden on healthcare systems, frequently requiring intensive care admissions, prolonged hospital stays, and increased resource utilization and costs with corresponding economic implications [21,22].

In Poland, comprehensive nationwide data are available through the PolPeDiab Study Group, which has been systematically collecting multicenter pediatric T1D data covering nearly the entire country for several years. These data indicate that Poland has a particularly high rate of DKA at the onset of T1D, with the prevalence varying across different regions. Before the COVID-19 pandemic, the rate of DKA was estimated between 27.9% (Lower Silesia) and 46.8% (West Pomeranian) in children up to 18 years of age. Additionally, during the pandemic, the DKA rate dramatically rose in all regions of Poland [23,24]. Studies conducted in Poland strongly suggest that misdiagnosis or delayed recognition of T1D symptoms at initial presentation contributes to the incidence of DKA. A lack of early suspicion among healthcare providers, combined with the nonspecific nature of early T1D symptoms and delayed caregiver recognition, often leads to missed opportunities for timely intervention [13,23,24].

Factors contributing to delayed diagnosis of T1D and progression to DKA are multifactorial, resulting from a combination of patient-, caregiver-, and healthcare system-related barriers. These include communication barriers in younger children, nonspecific or rapidly evolving symptoms, insufficient awareness of early warning signs, misattribution of symptoms, and limited access to diagnostic testing [25,26,27]. This conceptual categorization underlies the structure of the current review and informs the discussion of preventive strategies.

If not promptly recognized and treated, DKA can lead to serious complications such as cerebral edema and death [10,28]. Effective management of DKA in pediatric patients is particularly challenging due to the severity and overlap of systemic manifestations during acute metabolic decompensation, as well as the risk of rapid fluid shifts and electrolyte imbalances. Therefore, precise estimation of fluid and electrolyte deficits is critical, and careful monitoring is necessary to avoid complications during treatment.

This narrative review aims to enhance clinical practice by summarizing diagnostic and management algorithms for primary care clinicians, facilitating the early identification and effective management of newly diagnosed T1D in children and adolescents, ultimately improving patient outcomes and quality of care in this vulnerable population.

## 2. Materials and Methods

This narrative review was conducted without a systematic literature search. A narrative approach was selected to facilitate the inclusion of evidence from heterogeneous study designs, clinical guidelines, and real-world practice observations, which are particularly relevant to the clinical and educational foci of this article when addressing diagnostic challenges, red flag symptoms, and early management pathways in primary care. Each author independently selected and critically appraised relevant studies regarding DKA at the onset of T1D in children and adolescents. Discrepancies in study selection and interpretation were resolved through discussion and consensus among the authors. Given the narrative and practice-oriented nature of this review, no formal risk-of-bias or quantitative quality assessments were employed; however, priority was given to higher levels of evidence, such as international guidelines, systematic reviews, and large observational studies, particularly when informing clinical algorithms and recommendations.

The inclusion criteria were clinical studies, reviews, systematic reviews, and meta-analyses that discussed new-onset T1D and DKA in children and adolescents. Exclusion criteria included non-English language papers, studies with insufficient data, non-peer-reviewed articles, duplicated, unavailable full texts, or abstract-only papers. Articles were sourced from several databases, including PubMed, Google Scholar, EMBASE, Scopus, and Web of Science. Search period spanned from August 2024 to April 2025, and keywords used included: “type 1 diabetes,” “diabetic ketoacidosis,” “pediatric diabetic ketoacidosis,” “Polish population,” “children and adolescents,” “onset of type 1 diabetes,” “glycemic control,” “hyperglycemic crisis,” “diabetic acidosis,” “management of diabetic ketoacidosis,” “clinical features,” “risk factors,” “diabetic ketoacidosis at diagnosis,” “prevalence of diabetic ketoacidosis,” “frequency of diabetic ketoacidosis,” “diabetes care,” “screening programs”, “prevention”, among others. The term “Polish population” was included to reflect the authors’ clinical experience and to identify region-specific epidemiological and healthcare system data, illustrating diagnostic challenges and DKA prevalence patterns relevant to Central and Eastern Europe, while remaining contextual to the overall scope of this narrative review. Studies published between 2004 and 2025 were screened by title, abstract, and full text to identify the most clinically relevant works. However, some earlier landmark studies that fell outside of the stated publication window were considered due to their high relevance to the objectives of this narrative review and to ensure historical context is adequately captured. This selection aimed to provide theoretical insights and practical frameworks to enhance the recognition and diagnosis of DKA at the onset of T1D in pediatric patients, serving as an informative resource for clinical and educational improvement in pediatric diabetes care among practitioners.

## 3. Symptomatology and Clinical Onset

Children with early T1D often present symptoms that overlap with those of common pediatric conditions, leading to frequent misdiagnoses and delays in receiving appropriate care. Table 1 presents red flag symptoms, grouped into broader clinical categories, in children to assist primary care clinicians in their routine practice in recognizing both classic and atypical manifestations of hyperglycemia and DKA, warranting timely blood glucose testing. Based on a retrospective chart review of 276 patients aged 1 year to 17 years, despite the near-universal presentation of polyuria and polydipsia among children with new-onset T1D (91%), additional symptoms like weight loss (58%), abdominal pain (17%), and lethargy (13%) vary significantly, often complicating diagnosis and leading to misdiagnosis as other illnesses [29]. Symptoms such as increased thirst, frequent urination, nausea, vomiting, and fatigue can mimic various infections and gastrointestinal issues, often resulting in misdiagnoses, including gastroenteritis, respiratory infections, viral syndromes, or strep pharyngitis [30]. Dehydration, frequently diagnosed in children with early symptoms of T1D, is sometimes managed with fluid replacement alone, which does not address the underlying insulin deficiency, ultimately exacerbating DKA risk [29]. Additionally, symptoms like abdominal pain and vomiting are sometimes mistaken for other conditions, such as constipation or reflux. In some cases, infections (e.g., acute otitis media or oral thrush) or inflammatory responses (e.g., dermatitis, folliculitis) divert clinical attention, while conditions such as steroid-induced hyperglycemia can also complicate diagnosis [29].

This issue is pronounced in younger children, who face a higher risk of presenting with DKA at T1D onset, where symptoms can be challenging to identify, often presenting in subtle or nonspecific forms and complicated by communication challenges [31]. These signs may overlap with other pediatric conditions, such as asthma or bronchiolitis, leading to frequent misdiagnoses and treatments, like glucocorticoids or sympathomimetics, that can exacerbate metabolic imbalances [13]. Children under five, in particular, show an increased incidence of DKA compared to older age groups, underscoring the diagnostic difficulties within this demographic [17,24,32,33,34]. From a developmental and physiological perspective, younger children are more vulnerable to rapid metabolic decompensation due to limited glycogen reserves, higher basal metabolic rates, and limited capacity to compensate for insulin deficiency [35]. Moreover, their restricted ability to communicate symptoms, reliance on caregiver observation, and rapid transition from mild hyperglycemia to acidosis increase the likelihood of delayed diagnosis and severe DKA at presentation [25,36]. There is currently no standardized tool or point-based scoring system that is formally available to primary care physicians or emergency department clinicians to support early recognition of new-onset T1D. However, the current ISPAD guidelines recommend immediate glucose measurement in any child presenting with nonspecific symptoms such as dehydration, vomiting, tachypnea, or altered mental status [8]. In addition, international consensus statements suggest that combining glucose and ketone measurements in children with established T1D can help prevent progression to severe DKA [37]. Extending this approach to perform both glucose and ketone testing not only in children with known T1D but also in uncertain or high-risk situations, including non-communicative patients, could theoretically facilitate earlier recognition of T1D and DKA in primary care and emergency settings. However, no studies have yet evaluated whether such a strategy would directly reduce the incidence of DKA at diagnosis. Significantly, the detection of red flags in young children relies on targeted history obtained from legal guardians by a physician. This should routinely include questions about recent weight changes, particularly unintentional weight loss, as well as a thorough physical examination focused on identifying clinical signs of dehydration. Key features include delayed capillary refill, dry mucous membranes, reduced skin turgor, sunken eyes, tachycardia, and prolonged peripheral coolness. These findings are critical because dehydration is one of the principal clinical manifestations of DKA at the onset of T1D. Strengthening the clinical assessment in this manner is essential, particularly in children who cannot reliably express their symptoms [8].

Studies have shown that infections, both viral and bacterial, are frequently associated with an increased risk of DKA at the onset of T1D in children [38]. First, infections may act as potential metabolic precipitants, exacerbating autoimmune-mediated processes that damage pancreatic β-cells, leading to worsened metabolic control and a heightened risk of DKA [33,39,40]. This is attributed to the inflammatory response, characterized by the release of cytokines and an elevation in counter-regulatory hormones, which collectively promote insulin resistance and metabolic dysfunction [41]. Second, infections may serve as diagnostic distractors, masking T1D symptoms with overlapping symptoms such as vomiting, fatigue, or fever, potentially leading healthcare providers to misinterpret these signs as an acute illness and further delay appropriate diagnostic testing, thereby preventing a correct diagnosis. Distinguishing between infections of different roles is crucial, as both mechanisms could independently contribute to delayed diagnosis and increased risk of severe DKA at onset. Addressing this issue through heightened awareness and prompt recognition of T1D’s early warning signs could help reduce diagnostic delays and improve outcomes.

Table 2 summarizes the key contributors to delayed or missed diagnosis in children with newly diagnosed T1D, with factors grouped conceptually into symptom-related factors (e.g., atypical or misleading presentations), age-related factors (particularly in younger children), and clinician- or system-related factors that may result in misinterpretation or delayed testing.

## 4. Evaluation and Treatment of Hyperglycemia in Children and Adolescents: A Guide for Clinicians

Early recognition of hyperglycemia in children is vital for preventing severe complications and initiating timely management. Clinicians should carefully assess red flags for hyperglycemia, including classic symptoms such as polyuria/nocturia, polydipsia, and weight loss, as well as additional presenting symptoms, including abdominal pain, nausea, vomiting, lethargy, Kussmaul breathing, and confusion. The presence of persistent vomiting, abdominal pain, dehydration, Kussmaul breathing, or any degree of altered mental status should raise immediate concern for imminent or established DKA rather than uncomplicated hyperglycemia. DKA is a common initial presentation of T1D in children and may progress rapidly; therefore, delays in recognition or escalation of care significantly increase morbidity. In such cases, urgent evaluation with point-of-care glucose, ketone testing, and acid–base assessment is warranted, and management should not be delayed for confirmatory investigations. History taking may help reach the diagnosis and should include assessment of age, body mass index (BMI), BMI standard deviation score (SDS), pubertal status, recent illnesses, and a family history of T1D, type 2 diabetes (T2D), and autoimmune diseases [42]. Anthropometric features such as BMI, BMI SDS, and pubertal status can provide useful contextual information when considering diabetes subtype, as T2D is more likely in youth with overweight or obesity, particularly after the onset of puberty, whereas T1D more commonly presents with unintentional weight loss and acute onset in prepubertal children [42]. However, these clinical features are not definitive, particularly in the context of the current pediatric obesity epidemic, and must be interpreted in conjunction with biochemical findings. A structured history aids in identifying both genetic and environmental risk factors and may raise suspicion for alternative diagnoses such as monogenic diabetes in non-obese children with mild hyperglycemia and a strong autosomal dominant family history [42].

Importantly, biochemical testing for diabetes should be performed in all symptomatic children, even when symptoms are mild or a positive family history is present [42]. Clinical features and family history alone are insufficient to exclude diabetes and may result in false reassurance, as most children who develop T1D do not have a known affected relative [42,43]. Reliance on caregiver familiarity with early symptoms or on perceived low risk based on family history can therefore lead to missed or delayed diagnoses [43]. Current recommendations from major professional organizations emphasize that any child with symptoms suggestive of hyperglycemia, regardless of severity, should undergo laboratory evaluation, such as fasting plasma glucose, HbA1c, or OGTT, and that diagnosis and management should not be delayed when results are unequivocally diagnostic [42].

Early biochemical testing, combined with education about diabetes warning signs, has been shown to reduce the likelihood of DKA at presentation [43]. This is particularly important given the substantial overlap in clinical presentation between diabetes subtypes, including the increasing prevalence of obesity among children with T1D and the occurrence of T2D in younger and prepubertal patients [42]. Children with a known family history of T1D may present with milder metabolic decompensation due to heightened awareness and earlier recognition of symptoms, but such information should complement rather than delay timely biochemical confirmation and clinical action [43]. Moreover, routine inquiries into changes in thirst and urination patterns, combined with a low threshold for laboratory testing and integration of symptom prompts into electronic medical records, may facilitate earlier diagnosis and reduce the risk of DKA-related complications [29,38]. However, implementation in routine clinical practice may be limited by factors such as alert fatigue and variability across primary care systems [38]. One pragmatic approach is to incorporate brief symptom checklists or automated prompts into electronic medical records during routine pediatric visits, triggering capillary glucose testing when key red flags are present [38].

Moreover, routine inquiries into changes in thirst and urination patterns, combined with a low threshold for laboratory testing and integration of symptom prompts into electronic medical records, may facilitate earlier diagnosis and reduce the risk of DKA-related complications [29]. However, implementation in clinical practice may be limited by factors such as alert fatigue and variability across primary care systems. One pragmatic approach is to incorporate brief symptom checklists or automated prompts into electronic health records during routine pediatric visits, triggering capillary glucose testing when key red flags are present.

## 5. Initial Diagnostic Tests and Further Referral

Diagnosis of diabetes in the pediatric population is similar to that in adults, typically using fasting or random plasma glucose levels and/or glycated hemoglobin (HbA1c) levels, and depends on the presence or absence of symptoms. These diagnostic criteria are largely consistent across major international guidelines, including those of the International Society for Pediatric and Adolescent Diabetes (ISPAD), the American Diabetes Association (ADA), the Polish Diabetes Association (PTD), the World Health Organization (WHO), and the National Institute for Health and Care Excellence (NICE) [7,8,44,45,46,47]. The use of plasma glucose measurements, such as fasting, random, or during an oral glucose tolerance test (OGTT), in conjunction with clinical symptoms, is recommended to establish the diagnosis, with guidelines underscoring the importance of rapid assessment and timely referral in cases suspected of DKA.

According to the current guidelines, diabetes can be diagnosed based on one or more of the following tests: fasting plasma glucose test, in which a blood sample is taken after the child has fasted for at least 8 h. A fasting blood glucose level of 126 mg/dL (7.0 mmol/L) or higher indicates diabetes. While a random plasma glucose test means that a blood sample is taken at any time, regardless of when the child last ate. A level of 200 mg/dL (11.1 mmol/L) or higher suggests diabetes, especially if accompanied by symptoms. Additionally, if available, HbA1c testing is a valuable tool in diagnosing diabetes. This test measures the average blood glucose levels over the past 2 to 3 months. A level of HbA1c greater than 6.5% indicates diabetes [7]. While HbA1c ≥ 6.5% is accepted by the ADA as a diagnostic criterion, ISPAD emphasizes that HbA1c should not be used as the sole diagnostic test in symptomatic children, especially when rapid disease progression or DKA is suspected [8,45]. However, biochemical thresholds are unified, but clinical decision-making and urgency vary, especially in pediatric care. Some guidelines (e.g., NICE, ISPAD) prioritize symptom-based diagnosis and immediate treatment, while others (e.g., WHO) emphasize methodological caution. Table 3 presents differences in emphasis across diabetes guidelines [7,8,44,45,46,47].

In everyday practice, diagnosing hyperglycemia in a child presenting with diabetes symptoms in primary care is simple and inexpensive, using a simple finger-prick glucose test. The level of random plasma glucose levels determines further management. If a child presents with a random glucose level ≤ 125 mg/dL (≤6.9 mmol/L), the diagnosis of diabetes is unlikely. Then, the pediatrician should investigate other possible causes of the presented symptoms. However, if a random glucose level is equal to or higher than 200 mg/dL (11.0 mmol/L), a diabetes diagnosis should be presumed by primary care clinicians. In such cases, patients with significant hyperglycemia or any suspicion of DKA require immediate referral to an emergency department or pediatric endocrinology/diabetes unit for further management [16]. In a symptomatic child with classic features of hyperglycemia, a single elevated random plasma glucose value ≥ 200 mg/dL (≥11.1 mmol/L) is sufficient to warrant urgent referral and immediate clinical action, and confirmatory laboratory testing should not delay management. Current ADA and American Academy of Pediatrics (AAP) guidelines state that repeat testing is not required in the presence of unequivocal hyperglycemia with symptoms, as diagnostic delays increase the risk of DKA and worse outcomes in children [16].

**Figure 2:**
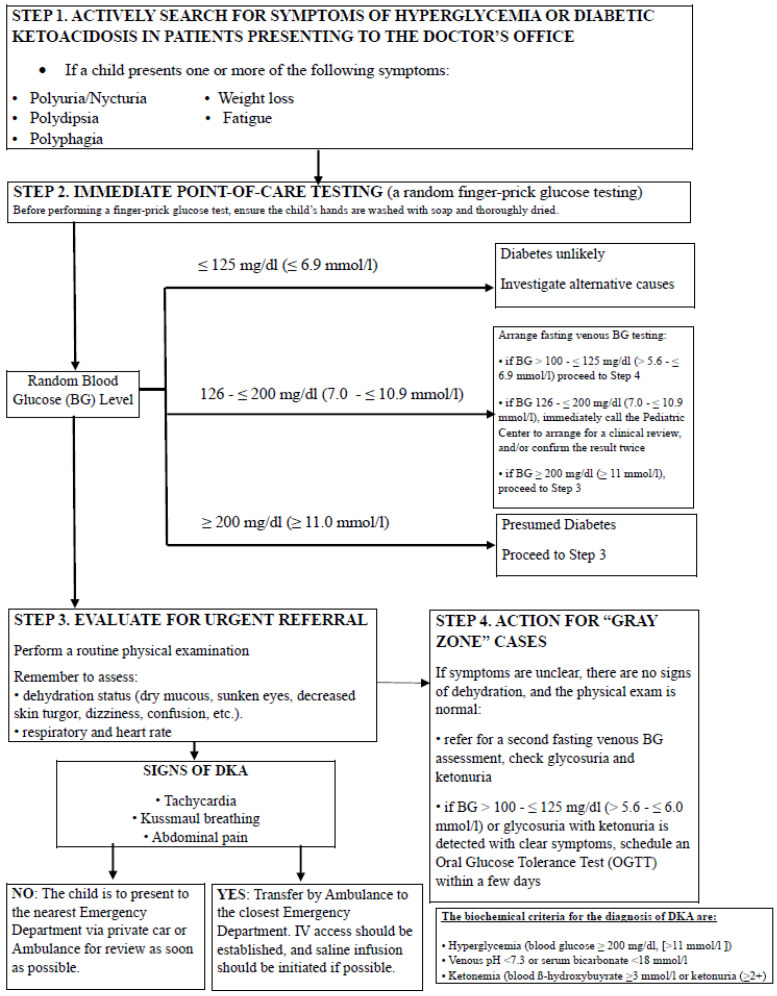
Practical diagnostic flowchart for primary care clinicians detailing initial symptom recognition, point-of-care glucose testing, and referral pathways for suspected type 1 diabetes and diabetic ketoacidosis in children and adolescents, adapted from ISPAD, ADA, and PTD guideline recommendations [7,8,44,45,46,47].

For individuals presenting with symptoms suggestive of diabetes but with normal fasting glucose levels or abnormal glucose levels not meeting the criteria of diabetes, an OGTT using a 1.75 g/kg glucose dose (up to 75 g) may provide additional information. OGTT is performed after fasting; the child drinks a glucose solution, and blood samples are taken at intervals to see how the body processes glucose. A 2 h plasma glucose level of 200 mg/dL (11.0 mmol/L) or higher indicates diabetes. The OGTT is not recommended in the acute diagnostic setting for children with significant hyperglycemia or suspected DKA. In symptomatic children (polyuria, polydipsia, weight loss) or those presenting with hyperglycemic crisis, a random plasma glucose ≥ 200 mg/dL (≥11.1 mmol/L) is sufficient for diagnosis and urgent referral, and treatment should not be delayed for confirmatory testing. OGTT should be reserved for diagnostically ambiguous, clinically stable pediatric patients, such as those with mild or equivocal hyperglycemia or discordant fasting glucose and HbA1c results. In these settings, particularly in children with obesity or risk factors for T2D, OGTT can help identify impaired glucose tolerance or confirm diabetes. Because OGTT requires fasting and is time-consuming, it is unsuitable for acutely ill or unstable children and should be used only when results will meaningfully guide diagnosis in non-acute settings [48]. Figure 2 offers a practical, guideline-informed diagnostic flowchart for primary care clinicians, outlining early recognition, initial diagnostic testing, and referral pathways for hyperglycemia, suspected T1D, and DKA in children and adolescents, in accordance with ISPAD, ADA, and PTD guidelines.

It is important to note, however, that in clinical practice, diabetes in children often presents more rapidly and progresses more quickly than in adults. This is due to a shorter preclinical phase and a more abrupt decrease in insulin production, particularly in T1D. As a result, children are frequently diagnosed with significant hyperglycemia and metabolic instability at an early stage [49]. Therefore, clinicians mustn’t delay glucose testing until fasting conditions can be met. Diagnostic strategies commonly used in adults, such as reliance on fasting glucose measurements or delayed testing strategies, may be unsafe in children due to the rapid progression to metabolic decompensation and increased risk of DKA. Random plasma glucose should be measured immediately when diabetes is suspected, regardless of the time of day or the child’s last meal. Capillary glucose measurement using a personal glucometer provides an effective initial diagnostic tool in resource-limited or outpatient settings. This rapid assessment enables timely clinical decision-making and facilitates appropriate referral to specialized care if necessary. Incorporating this practice into routine pediatric assessments is crucial for early diagnosis and the prevention of complications, such as DKA [50]. For children with symptoms suggestive of DKA, an immediate diagnostic workup in the emergency department should be performed. This typically includes measuring venous blood glucose levels, blood gases, and urine ketones. The severity of DKA should be classified based on pH and bicarbonate levels: mild (pH < 7.3, bicarbonate < 18 mmol/L), moderate (pH < 7.2, bicarbonate < 10 mmol/L), and severe (pH < 7.1, bicarbonate < 5 mmol/L). This classification guides immediate management and the urgency of referral. Clinicians should also check for elevated serum osmolality (>290 mOsm/L) and calculate the anion gap, which is typically >10 mEq/L in DKA [12]. After confirming the new onset of diabetes, a doctor should contact the nearest pediatric diabetes care center to arrange a referral and transfer of the patient.

## 6. Emergency and Ambulance Management Steps

To prevent rapid deterioration and minimize complications in pre-hospital and emergency department (ED) settings, immediate blood gas analysis is crucial to assess the patient’s metabolic status, including pH, bicarbonate levels, and the anion gap, which are key indicators of DKA severity. In pre-hospital or ambulance settings, initial assessment should prioritize rapid clinical evaluation, recognition of signs suggestive of DKA, and point-of-care capillary glucose measurement [51,52,53]. More detailed diagnostic investigations, including blood gas analysis, serum electrolytes, and ketone assessment, are typically deferred until arrival in the emergency department, where appropriate resources and monitoring are available. In critically ill children, immediate clinical stabilization—including airway protection, circulatory support, and cautious fluid resuscitation—should take precedence over extensive laboratory testing, which should not delay urgent life-saving interventions. Additionally, urine tests for ketones should be conducted to confirm the diagnosis of DKA. Alongside blood gas and urine tests, a basic laboratory workup should include a complete blood count (CBC), electrolyte levels (sodium, potassium, chloride), blood glucose measurement, and kidney function tests, such as serum creatinine and blood urea nitrogen (BUN). These tests provide essential information for evaluating the overall metabolic state and guide subsequent treatment decisions.

Hydration is a cornerstone of DKA management. The ISPAD guidelines recommend administering intravenous (IV) fluids to correct dehydration and restore circulatory volume [8]. An initial fluid regimen typically involves isotonic saline (0.9% sodium chloride) at a rate of 10–20 mL/kg/hr during the first hour, adjusted according to the patient’s degree of dehydration and clinical condition. Ongoing fluid therapy should be titrated based on the patient’s fluid balance, renal function, and electrolyte levels. While bicarbonate in the pediatric population is generally not recommended as routine treatment. The use of bicarbonate can lead to rapid shifts in potassium levels, increasing the risk of life-threatening arrhythmias [7]. Although current international guidelines provide general recommendations for fluid management in pediatric DKA, ongoing debate remains regarding optimal fluid rates and the risk of cerebral edema; therefore, local protocols, institutional guidelines, and patient-specific factors should guide final clinical decisions.

After initial stabilization, contact the nearest pediatric diabetes center immediately to arrange the patient’s transfer and continue management according to the guidelines and other relevant national pediatric diabetes care protocols. These guidelines provide detailed recommendations for insulin therapy, electrolyte management, and monitoring of blood glucose levels until the patient reaches clinical stability [8]. Incorporating these management steps into pre-hospital and emergency department protocols, as emphasized in the ISPAD Guidelines, enhances early detection and treatment of pediatric DKA, promoting timely intervention, reducing the risk of complications, and improving patient outcomes.

Early involvement of a pediatric diabetes center supports standardized insulin initiation, expert monitoring for acute complications such as cerebral edema, and seamless continuity of care following diagnosis. Multidisciplinary teams apply protocol-driven insulin regimens, provide structured education, and ensure close early follow-up, which together improve safety, reduce acute complications and hospitalizations, and are associated with better glycemic outcomes and quality of life for children with newly diagnosed T1D [8].

While population-wide screening programs for T1D offer clear benefits in reducing the incidence and severity of DKA at diagnosis, their implementation raises important practical and ethical considerations. These include financial costs, the need for adequate healthcare infrastructure, and the potential psychological burden associated with identifying presymptomatic disease in children and families. Addressing these challenges requires appropriate counseling, structured follow-up pathways, and careful integration of screening initiatives into existing healthcare systems to ensure that the benefits of early detection outweigh potential harms.

## 7. The Strengths and Limitations of the Review

The strength of this narrative review lies in its practical summarization of the available knowledge on initial symptoms and early management of T1D in the pediatric population. This review is specifically intended for frontline clinicians, including general practitioners, pediatricians, and emergency department clinicians, who are most likely to encounter children with early or atypical presentations of T1D and are therefore pivotal in preventing diagnostic delays and DKA. The work highlights relevant causes responsible for T1D misdiagnosis. Additionally, we provide a general overview of common strategies and programs implemented to reduce the rate of DKA at the onset of T1D. These strengths are reinforced by the practical tools presented earlier in the manuscript, including structured symptom tables and diagnostic algorithms, which translate heterogeneous evidence into actionable clinical guidance and partially compensate for the absence of a systematic methodology.

The main limitation of this paper is its general summarization of published guidelines. As a narrative review, this work is also subject to inherent limitations, including potential selection bias and the absence of a formal systematic search strategy or structured quality appraisal, which may influence the weighting of included evidence. For more information, detailed guidelines, and official statements, doctors and clinicians should refer to national or international recommendations, such as the ISPAD 2022 Clinical Practice Consensus Guidelines [7,8]. However, we hope that our work will serve as a valuable tool for primary care clinicians, offering practical guidance in everyday medical practice to enhance early detection and intervention.

## 8. Clinical Implications

The findings underscore the critical need for improved diagnostic vigilance among healthcare providers to mitigate the high burden of DKA at the onset of T1D in children and adolescents. Misdiagnoses stemming from overlapping symptomatology with common pediatric conditions often delay appropriate interventions, heightening the risk of severe metabolic complications. Structured diagnostic algorithms and heightened awareness of atypical presentations, particularly in younger children, can facilitate earlier recognition of hyperglycemia and DKA. Systematic family history assessments and population-wide screening programs for autoantibodies and genetic predispositions may enhance early detection. These measures are crucial in reducing DKA incidence, particularly among high-risk groups, and enhancing outcomes by facilitating timely therapeutic interventions.

Population-wide screening programs for T1D have been shown to reduce both the incidence and severity of DKA at diagnosis, particularly when islet autoantibody testing is combined with education and structured follow-up [45,54,55]. Large population-based studies demonstrate markedly lower DKA rates in screened cohorts compared with unscreened populations; however, implementation raises important practical and ethical considerations. These include financial costs, cost-effectiveness, healthcare infrastructure requirements, and the potential psychological impact on children and families, such as anxiety related to presymptomatic disease identification [55,56,57,58]. Current guidelines, therefore, emphasize informed consent, appropriate psychological support, and integration of screening into structured follow-up pathways within existing healthcare systems to ensure that the benefits of early detection are realized while minimizing potential harms [45,54,55,58,59].

GPs play a crucial role in reducing and addressing DKA at the onset of T1D by maintaining a high level of suspicion for T1D in children presenting with red flag symptoms, which is integral to improving patient outcomes and long-term quality of life. Improved GP education and diagnostic awareness are associated with shorter time to diagnosis, milder metabolic decompensation at presentation, and reduced severity of DKA, ultimately lowering the need for intensive care and improving early disease trajectories. Fulfilling this role requires regular, structured professional development focused on the early recognition of T1D, thus maintaining clinical proficiency in pediatric diabetes care. Educational campaigns targeting both healthcare providers and the general public, combined with the integration of standardized diagnostic tools, are crucial in reducing diagnostic delays. Public health strategies should emphasize early recognition of symptoms such as polyuria, polydipsia, and unexplained weight loss, alongside the importance of monitoring high-risk children. Leveraging advancements in screening technologies and refining clinical protocols can significantly reduce the prevalence of DKA at diagnosis, transforming the trajectory of T1D care and ensuring better health outcomes for affected children.

Overall, this review highlights that early recognition of red flag symptoms, supported by targeted GP education, structured diagnostic pathways, and the implementation of appropriate screening strategies, represents the most effective approach to reducing DKA at the onset of T1D and improving outcomes for children and adolescents.

## 9. Initiatives to Reduce DKA at T1D Onset, Screening Programs to Lower DKA Incidence, and Future Directions

Educational initiatives aimed at raising awareness about the early signs and prevention of DKA in T1D have had varying levels of success across different countries and regions (Table 4). Successful campaigns, such as those by Holder & Ehehalt, Vanelli et al., and King et al., have effectively used public education programs and media campaigns through posters, leaflets, toll-free hotlines, and digital platforms to raise awareness of T1D symptoms [42,60,61,62,63]. Public campaigns in Germany, Saudi Arabia, and Italy have notably reduced DKA rates through sustained outreach and culturally relevant materials, emphasizing the importance of early detection and prompt intervention [60,61,64].

The successful reduction in DKA rates through public education is further supported by targeted healthcare provider training, which equips clinicians with glucose and ketone testing meters and standardized diagnostic protocols, thereby improving early diagnosis and management of DKA [62,63,65]. However, sustainability in education and continuous professional development remain a challenge, as highlighted by Darmonkow et al. Improvements often decline once the program concludes, emphasizing the need for ongoing education and up-to-date materials [66].

Conversely, campaigns with mixed outcomes, such as the nationwide initiative in Italy, reveal the limitations of broader, less targeted interventions. While some improvements were noted in preschool-aged children (under 6 years of age), the overall DKA rates increased among other groups and older cohorts, highlighting the challenges in achieving a widespread impact across all demographics [67]. This inconsistency underscores the challenges of reaching a diverse population and maintaining engagement across various media channels, despite the increasing use of standardized treatment protocols [71]. Localized efforts, such as those in Parma, have yielded more robust results, highlighting the importance of tailored interventions targeting specific communities or healthcare providers [61].

Campaigns in New Zealand, Austria, and Wales highlight critical pitfalls. A low recall rate, static educational materials without follow-up or diverse outreach methods, a short campaign duration, and insufficient healthcare engagement all contributed to unchanged or worsening DKA rates [68,69,70]. In particular, Austria’s reliance on GPs without specialized training in pediatric diabetes care and the sporadic implementation and fragmented approach in Wales underscore the risks of poorly structured initiatives [69,70]. Moreover, GPs may encounter organizational challenges, such as time and resource limitations. GPs often have limited consultation time, and limited access to point-of-care testing or laboratory facilities within the GP office can hinder prompt diagnosis. Another issue is a lack of financial support for campaigns or educational efforts about T1D, which can contribute to an increased rate of DKA at diagnosis. When GPs do not have adequate access to resources, education, or support services, they may be less likely to recognize early symptoms of T1D. Finally, cultural barriers can significantly impact the misdiagnosis of T1D in a GP’s office. These barriers may influence the recognition of symptoms and the communication between patients and healthcare providers. Addressing these obstacles requires increased awareness, education, and the establishment of protocols for early detection, particularly in children presenting with red flag symptoms [72,73].

Future campaigns should prioritize sustainability through continuous and targeted engagement, digital outreach (e.g., teleconsultations and mobile platforms), and regular updates and consistent reinforcement tailored to both the public and healthcare providers [42,63,64]. Collectively, these cases underscore the need for multi-layered, culturally sensitive interventions that integrate public education with targeted professional training to achieve impactful and lasting reductions in DKA at T1D onset [64,74].

While efforts in educational initiatives play a role in reducing DKA at T1D onset, screening programs—both population-based and risk-targeted—show a more robust impact in lowering DKA rates and facilitating early diagnosis. The TEDDY (The Environmental Determinants of Diabetes in the Young) study provides a comprehensive understanding of T1D progression from genetic predisposition to clinical onset. Longitudinal tracking of children with high genetic risk demonstrates that the presence of multiple autoantibodies—such as glutamic acid decarboxylase (GADA), tyrosine phosphatase-like protein IA-2 (IA-2A), and autoantibodies to insulin (IAA)—is strongly associated with a more aggressive disease course, particularly in children under two years of age [75]. Genetic predisposition, particularly high-risk *HLA* genotypes and a family history of T1D, plays a central role, with age influencing the development of autoantibodies [75]. These findings highlight the need for dynamic, age-tailored screening protocols.

Geographic variations in T1D risk, such as the high incidence in Finland compared to more stable rates in the USA and Sweden, emphasize the influence of localized environmental factors and the need for geographically tailored public health strategies [76]. Intensive, longitudinal monitoring in TEDDY among high-risk populations resulted in a lower incidence of DKA and earlier detection compared to community diagnoses, demonstrating the effectiveness of regular glucose and HbA1c monitoring, while integrating systematic screening and monitoring into public health policies [77].

Complementary research from the Sardinian and Swedish cohorts reinforces the predictive value of multi-autoantibody screening. In Sardinia, combinations such as islet-cell antibodies (ICA) and IA-2A achieved high specificity and positive predictive value for T1D risk [78]. In the Swedish cohort, thyroid autoimmunity, which correlated strongly with GADA and Zinc Transporter 8 Antibodies (ZnT8), is more prevalent among children with islet autoimmunity and exhibits gender-specific patterns, emphasizing the need for comprehensive autoimmune screening in high-risk groups [79].

Despite these advances, TEDDY also identifies key challenges. The high cost and limited scalability of intensive monitoring to larger populations, the focus on *HLA*-defined high-risk groups, and the lack of approved therapies for younger children highlight the need for broader, more inclusive screening strategies and treatment development [80]. TEDDY findings emphasize the importance of enhancing screening models by incorporating additional biomarkers, such as C-peptide levels and progression likelihood scores, to further refine risk stratification and improve screening effectiveness [81]. Results from TEDDY and existing tools suggest the need to integrate cost-effective, multi-marker screening programs into public health strategies, enabling the earlier identification of individuals at risk, reducing the incidence of DKA, and preventing the progression of autoimmune diseases.

## 10. Summary

Primary care clinicians play a crucial role in the early identification of T1D in the childhood population; however, diagnostic delays remain common due to nonspecific symptoms and overlap with routine pediatric illnesses. A clear understanding of DKA pathophysiology, early metabolic changes, and typical symptom patterns can support more confident and timely clinical decision-making. In everyday practice, when children present with unclear or nonspecific symptoms, point-of-care finger-prick glucose measurement remains the most appropriate and practical initial screening tool, allowing clinicians to rapidly assess for hyperglycemia. Strengthening referral pathways and ensuring rapid access to specialist assessment further improve safety at the time of diagnosis. Consistent use of structured protocols and educational initiatives provides a realistic path toward reducing DKA incidence and improving outcomes for children and adolescents with new-onset T1D.

## Figures and Tables

**Table 1:** Red flag symptoms in children that warrant blood glucose testing [5,6,7,8,29,30].

Classic Hyperglycemia Symptoms	Excessive Thirst and Dry Mouth (Polydipsia)
	Frequent Urination (Polyuria)
	Unintentional Weight Loss
	Increased Hunger (Polyphagia)
General and constitutional symptoms	Fatigue and weakness
	Blurred vision
Gastrointestinal manifestations	Nausea and vomiting
	Abdominal pain
Respiratory and metabolic signs	Sweet or fruity-smelling breath (acetone smell)
	Rapid breathing or shortness of breath
Neurological symptoms	Confusion or altered mental state
	Lethargy or unresponsiveness
Signs of dehydration	Skin changes: dry, flushed skin
	Dry mucous membranes, sunken eyes

**Table 2:** A summary of symptom-, age-, and clinical-related diagnostic difficulties contributing to delayed or missed diagnosis at childhood type 1 diabetes onset [5,6,7,8,13,31].

Symptom/Characteristic Feature	Reason for Late Diagnosis
**Young age**	Children under 4 years of age may present symptoms typical of hyperglycemia, but parents may not recognize them unless actively questioned. Additionally, obtaining a medical history from a child can be challenging, as it requires doctors to rely heavily on parental observations. These factors may lead to a late diagnosis.
**The hyperventilation of DKA**	It may be misdiagnosed as asthma exacerbations or pneumonia/bronchiolitis (cough and breathlessness distinguish these diseases from DKA), which are, in fact, Kussmaul respirations. Commonly, antibiotics or GKS are initiated, especially GKS, which can exacerbate the severity of hyperglycemia.
**Vomiting without** **diarrhea**	Typically, a child presenting with vomiting is inappropriately diagnosed with viral gastroenteritis.
**Abdominal pain**	It is related to DKA, and it may simulate an acute abdomen, such as appendicitis, which can result in a referral to a surgeon.
**New bedwetting**	Nocturia and bedwetting in a previously potty-trained child are often assumed to be psychogenic. These symptoms are flashing warning signs to investigate further before classifying them as psychogenic.
**Polyuria**	In most cases, children with polyuria and enuresis are misdiagnosed with UTI because of increased frequency of urination. Sometimes, antibiotics are started without performing a urine analysis.
**Polydipsia**	Although this is the most obvious sign of diabetes, increased thirst can easily be blamed on hot weather (especially in summer months), or it can also be considered psychogenic.
**Weight loss**	Another typical symptom, however, some pediatricians related it to growth spurts, increased activity with a new sport, or an intentional lifestyle change.

DKA—diabetic ketoacidosis; GKS—glucocorticoids; UTI—urinary tract infections.

**Table 3:** Differences in emphasis across diabetes guidelines [7,8,44,45,46,47].

Organization	Differences/Emphasis
**ISPAD**	Pediatric-specific; recommends autoantibodies and C-peptide for classification; HbA1c is not the sole criterion in acute onset.
**ADA**	Uses standard thresholds; emphasizes prompt treatment in symptomatic children; recommends autoantibody testing.
**PTD (Poland)**	Adopts WHO/ISPAD thresholds; stresses early diagnosis and use of classification tools (autoantibodies, C-peptide).
**WHO**	Global standards highlight the limitations of HbA1c in the context of anemia, hemoglobinopathies, and acute illness.
**NICE (UK)**	Prioritizes clinical presentation: glucose ≥ 200 mg/dL with symptoms is sufficient; recommends immediate referral and initiation of insulin.

ISPAD—International Society for Pediatric and Adolescent Diabetes; ADA—American Diabetes Association; PTD—Polish Diabetes Association; WHO—World Health Organization; NICE—National Institute for Health and Care Excellence; UK—United Kingdom; HbA1c—glycated hemoglobin.

**Table 4:** A summary of key outcomes from awareness campaigns and screening initiatives, highlighting transferable strategies, implementation considerations, and lessons learned that may inform the design of future interventions aimed at reducing diabetic ketoacidosis at the onset of type 1 diabetes.

Outcome	Key Points
**Successful**	**Public Education Programs and Media Campaigns**: Utilize posters, leaflets, toll-free hotlines, and digital platforms to target the general public, families, and healthcare professionals. Significant reductions in DKA rates, e.g., 65% in regions like Germany, Italy, and Saudi Arabia, have been demonstrated [42,60,61,64]. **Healthcare Provider Training**: Targeted programs for clinicians improved early T1D diagnosis through tools like glucose meters and ketone tests. Focus on continuous professional development [62,65]. **Future Strategies**: Integrating digital tools, mobile apps, and local campaigns targeting high-risk groups (children, rural populations). Emphasis on sustained education and follow-up interventions [42,64,66].
**Mixed**	**Public Education Programs and Media Campaigns**: The Italian national campaign (2015–2017) utilized posters, newsletters, a bimonthly magazine, social media, and a commercial featuring famous comedians, addressing family pediatricians, families, and schools. It had inconsistent reach and effects on different cohorts. **Healthcare Provider Training**: Advertising posters and a monthly newsletter were sent via email to family pediatricians, who were the primary target of the awareness campaign. A survey of pediatric centers showed mixed results; DKA rates decreased in preschool children but increased in older age groups. **Future Strategies**: Emphasis on multi-pronged approaches that combine localized efforts, digital media campaigns, and innovative communication tools to sustain impact and ensure equitable access. Collaboration with family pediatricians, GPs, scientific societies, and national health systems is essential for reinforcing awareness campaigns and implementing effective nationwide DKA prevention initiatives [67].
**Failed**	**Public Education Programs and Media Campaigns**: Limited effectiveness in New Zealand and Austria, where campaigns relied primarily on posters and pamphlets, with low recall and unchanged DKA rates [68,69]. **Healthcare Provider Training**: Austria’s lack of specialized training in pediatric diabetes care for healthcare providers, primarily family doctors or GPs, hindered the campaign’s success, while Wales’s lack of follow-up contributed to its failure [69,70]. **Future Strategies**: There is a need for comprehensive, sustained outreach combining diverse media with professional education and regular reinforcement. Culturally sensitive, localized campaigns with follow-up interventions to maintain awareness and improve outcomes [69,70].

DKA—diabetic ketoacidosis; T1D—type 1 diabetes; GP—general practitioner.

## Data Availability

No new data were created or analyzed in this study. Data sharing is not applicable to this article.

## Abbreviations

The following abbreviations are used in this manuscript:

T1D	Type 1 diabetes
DKA	Diabetic ketoacidosis
BMI	Body mass index
SDS	Standard deviation score
T2D	Type 2 diabetes
HbA1c	Glycated hemoglobin
OGTT	Oral glucose tolerance test
ED	Emergency department
CBC	Complete blood count
BUN	Blood urea nitrogen
IV	Intravenous
GP	General practitioner
TEDDY	The Environmental Determinants of Diabetes in the Young
GADA	Glutamic acid decarboxylase
IA-2A	Insulinoma-associated protein 2
IAA	Insulin autoantibodies
ICA	Islet-cell antibodies
ZnT8	Zinc transporter 8 antibodies
ISPAD	International Society for Pediatric and Adolescent Diabetes
ADA	American Diabetes Association
AAP	American Academy of Pediatrics
PTD	Polish Diabetes Association
CVB	Coxsackievirus B
EBV	Epstein–Barr Virus
CMV	Cytomegalovirus
*HLA*	Human Leukocyte Antigen
IL2RA	Interleukin-2 Receptor Alpha
PTPN22	Protein Tyrosine Phosphatase Non-Receptor Type 22
CTLA-4	Cytotoxic T-Lymphocyte Antigen 4
FFA	Free fatty acid
Acetyl-CoA	Acetyl coenzyme A
WHO	World Health Organization
NICE	National Institute for Health and Care Excellence
